# In Vivo Assessment of the Ameliorative Impact of Some Medicinal Plant Extracts on Lipopolysaccharide-Induced Multiple Sclerosis in Wistar Rats

**DOI:** 10.3390/molecules27051608

**Published:** 2022-02-28

**Authors:** Rabia Rasool, Inam Ullah, Samiah Shahid, Bismillah Mubeen, Syed Sarim Imam, Sultan Alshehri, Mohammed M. Ghoneim, Sami I. Alzarea, Bibi Nazia Murtaza, Muhammad Shahid Nadeem, Imran Kazmi

**Affiliations:** 1 Institute of Molecular Biology and Biotechnology (IMBB), The University of Lahore, Lahore 54000, Pakistan; rabia.amjad545499@gmail.com (R.R.); inamgenetics@gmail.com (I.U.); samiah.shahid@imbb.uol.edu.pk (S.S.); bismillah.mubeen@gmail.com (B.M.); 2 Department of Pharmaceutics, College of Pharmacy, King Saud University, Riyadh 11451, Saudi Arabia; simam@ksu.edu.sa (S.S.I.); salshehri1@ksu.edu.sa (S.A.); 3 Department of Pharmacy Practice, College of Pharmacy, AlMaarefa University, Ad Diriyah 13713, Saudi Arabia; mghoneim@mcst.edu.sa; 4 Department of Pharmacology, College of Pharmacy, Jouf University, Sakaka 72341, Aljouf, Saudi Arabia; samisz@ju.edu.sa; 5 Department of Zoology, Abbottabad University of Science and Technology (AUST), Abbottabad 22310, Pakistan; nazia.murtaza@gmail.com; 6 Department of Biochemistry, Faculty of Science, King Abdulaziz University, Jeddah 21589, Saudi Arabia

**Keywords:** multiple sclerosis, oxidative stress, *Nepeta hindustana*, *Vitex negundo*, *Argemone albiflora*, neuroprotective agents

## Abstract

Multiple sclerosis is a chronic autoimmune disorder that leads to the demyelination of nerve fibers, which is the major cause of non-traumatic disability all around the world. Herbal plants *Nepeta hindustana* L., *Vitex negundo* L., and *Argemone albiflora* L., in addition to anti-inflammatory and anti-oxidative effects, have shown great potential as neuroprotective agents. The study was aimed to develop a neuroprotective model to study the effectiveness of herbal plants (*N. hindustana*, *V. negundo*, and *A. albiflora*) against multiple sclerosis. The in vivo neuroprotective effects of ethanolic extracts isolated from *N. hindustana*, *V. negundo*, and *A. albiflora* were evaluated in lipopolysaccharides (LPS) induced multiple sclerosis Wistar rat model. The rat models were categorized into seven groups including group A as normal, B as LPS induced diseased group, while C, D, E, F, and G were designed as treatment groups. Histopathological evaluation and biochemical markers including stress and inflammatory (MMP-6, MDA, TNF-α, AOPPs, AGEs, NO, IL-17 and IL-2), antioxidant (SOD, GSH, CAT, GPx), DNA damage (Isop-2α, 8OHdG) as well as molecular biomarkers (RAGE, Caspase-8, p38) along with glutamate, homocysteine, acetylcholinesterase, and myelin binding protein (MBP) were investigated. The obtained data were analyzed using SPSS version 21 and GraphPad Prism 8.0. The different extract treated groups (C, D, E, F, G) displayed a substantial neuroprotective effect regarding remyelination of axonal terminals and oligodendrocytes migration, reduced lymphocytic infiltrations, and reduced necrosis of Purkinje cells. The levels of stress, inflammatory, and DNA damage markers were observed high in the diseased group B, which were reduced after treatments with plant extracts. The antioxidant activity was significantly reduced in diseased induced group B, however, their levels were raised after treatment with plant extract. Group F (a mélange of all the extracts) showed the most significant change among all other treatment groups (C, D, E, G). The communal dose of selected plant extracts regulates neurodegeneration at the cellular level resulting in restoration and remyelination of axonal neurons. Moreover, 400 mg/kg dose of three plants in conjugation (Group F) were found to be more effective in restoring the normal activities of all measured parameters than independent doses (Group C, D, E) and is comparable with standard drug nimodipine (Group G) clinically used for the treatment of multiple sclerosis. The present study, for the first time, reported the clinical evidence of *N. hindustana*, *V. negundo*, and *A. albiflora* against multiple sclerosis and concludes that all three plants showed remyelination as well neuroprotective effects which may be used as a potential natural neurotherapeutic agent against multiple sclerosis.

## 1. Introduction

Multiple sclerosis (MS) is a neurodegenerative autoimmune disease, which is characterized as episodes of inflammation, neurons demyelination, and gliosis, with distinctive neuronal loss. It may be relapse-remitting or sometimes, progressive [1,2]. The pathophysiology demonstrates inflammatory lesions resulting in the development of several plaques in the white and grey matter of the spinal cord and brain [3]. Consequently, MS is known as the utmost reason for the neuronal disability that may disturb various systems in the body, resulting in countless neurological symptoms along with several comorbidities. These include visual disturbance, ataxia, sensory loss, double-vision, impaired balance, and muscle-weakness and affect the life expectancy [4]. MS may typically affect 2.5 million people over the globe [5]. The clinical manifestations vary from acute to chronic disease and entail lifestyle modifications. The edematous lesions form initially with perivascular cuffing with mononuclear cell infiltrations, broadly T-helper cells, and activated macrophages that infiltrate the white matter part of the brain afterward [6]. The blood–brain-barrier (BBB) and various inflammatory markers might get compromised; however, the vessel wall remains less vulnerable [7]. The myelin sheath has an abundance of proteins such as myelin basic proteins (MBP), which have a programmatic role in cytoskeletal turnover and regulate the structural integrity associated with myelin. Any alteration in immunological and enzymatic activity could conciliation the structural integrity and demyelination of neurons [8,9]. The treatment of multiple sclerosis is very challenging as it includes many drugs that could act via diverse mechanisms depending upon the severity of the disease as well as clinical courses. Up till now, there is no definite therapy that may impede disease progression at initial stages. However, the Food and Drug Administration (FDA) and European Medicines Agency (EMA) have approved Alemtuzumab, dimethyl fumarate, glatiramer-acetate, pegylated interferon-β, and vitamin-D for treatment of MS [10,11,12,13,14]. None of them is found to be efficacious in providing definite treatment or reversing of disease. The use of herbs in therapeutics has gained a lot of interest in the past few decades. Due to their antioxidant, anti-inflammatory, and distinctive potential, the use of phytochemicals will become a promising alternative for treating many neurodegenerative diseases as compared to current therapies [15].

*Nepeta hindustana* L. has been reported for its beneficial effects against hyperlipidemia, hypercholesterolemia, dyslipidemia, anti-phlogistic, anti-inflammatory, central nervous system (CNS) depressant, and sedative activities. The other genus of this family has been reported to have anti-Alzheimer activity [16,17,18]. *Vitex negundo* L., a member of *Verbenaceae*, is found in East Africa, India, The Philippine Island, Malaysia, and the warm zone of Taiwan. This aromatic shrub has been studied extensively due to its abundant pharmacological properties such as anti-inflammatory, anti-convulsant, and antinociceptive activities [19,20]. The phytoconstituents like vitexin protect dopaminergic neurons [21]. The leaves extract may improve cognitive impairment in rats by inhibiting lipid peroxidation, decreasing inhibitory acetylcholinesterase in the brain, and stimulating hair growth. It may also be beneficial for asthma, bronchitis, biliousness, spleen enlargement, eye disease, leucoderma, inflammation, and painful teething in infants [22,23]. *Argemone albiflora* L. is a flowering plant that belongs to the *Papaveraceae* family, localized in India, the United States, Ethiopia, and Bangladesh [24]. Due to analgesic, antispasmodic, anti-parasitic, and narcotic effects, it has been reported for its uses in many infectious diseases, malaria, dropsy, icterus, leprosy, warts, and jaundice. These activities are due to the secondary metabolites and some protein dissolving substances [25,26]. The effect of the selected plants used in the present research has been previously reported by Khare (2007), Anarthe and Chaudhari, (2011) and Rahiman et al. (2015) in Alzheimer’s and Parkinson’s [27,28,29]. However, their effect on MS has not been reported yet according to the best of our knowledge and literature. Hence, the current research is aimed to investigate the role of specific neuroinflammatory and oxidative stress biomarkers involved in the progression of the experimental autoimmune encephalomyelitis (EAE) Wistar rat model of neurodegeneration and to evaluate the neuromodulatory effects of ethanolic extracts from *N. hindustana*, *V. negundo*, and *A. albiflora* in treating neurodegeneration.

## 2. Materials and Methods

### 2.1. Chemicals

Lipopolysaccharide (*E.coli* serotype 0111:B4) (Sigma-Aldrich, St. Louis, MO, USA), chemicals, and reagents used in the present research were of analytical grade. All required buffers were prepared in the laboratories of the Institute of Molecular Biology and Biotechnology (IMBB), The University of Lahore, Pakistan. 

### 2.2. Experimental Animals

Wistar rats used in the experiment were bred under standard laboratory conditions. Animals were maintained as per national guidelines. All experimental work was performed according to the approved standard (IMBB/BBBC/21/1240) by the Departmental Biosafety and Biosecurity and Ethical Review Committee, The University of Lahore. 

### 2.3. Collection of Plant Materials 

The selected plants (*N. hindustana*, *V. negundo*, and *A. albiflora*) for the present research were collected locally from different areas of the Punjab, Pakistan, authenticated by the taxonomist, and were submitted with voucher numbers IMBB.UOL. 924-6, IMBB.UOL. 924-7, IMBB.UOL. 924-8. 

### 2.4. Preparation and Extraction of Plant 

The ethanolic extracts from selected plants were prepared using the modified method reported by Anokwuru et al. [30]. All extracts were filtered and evaporated using a rotavapor at 40 °C. Thereafter, the three collected ethanolic extracts were lyophilized. The percentage yield of all three plants was found as 8.12% for ethanolic extract of *N. hindustana*, 5.62% of *V. negundo*, and 41.5% for *A. albiflora* by

% yield = (Extract/plant material) × 100

### 2.5. Induction of Neurotoxicity

The rats were divided into seven groups named (A, B, C, D, E, F, G) and each group was further composed of five rats. Group A was taken as a control while the remaining (B, C, D, E, F, G) were injected with lipopolysaccharide (LPS) prepared in normal saline, 15 µg/rat. The LPS was injected bilaterally into the lumbosacral region of the spinal cord for three weeks to induce neurodegeneration (neurotoxicity) according to a modified protocol of Felts et al. [31] and Lassman et al. [32]. After the appearance of clinical signs and symptoms of neurodegeneration such as hind limb and tail paralysis, lethargy, fur tarnishing, weight loss, groups C, D, E, and F, were given plant extract treatment, and group G was delivered standard drug nimodipine for 28 days intraperitoneally according to Table 1. Nimodipine is a drug used in the treatment of multiple sclerosis, as it restores spinal oxygenation, improves function rapidly, and reduces demyelination in EAE as well as early MS lesion model [33]. At the end of the experimental protocol, all groups were dissected and the brain, liver, and spinal cord were taken for histopathological analysis. Blood and serum samples were also collected from all experimental rats.

### 2.6. Biochemical Estimation

#### 2.6.1. Estimation of Super Oxide Dismutase (SOD)

Superoxide dismutase (SOD) activity was determined via the method of Kakkar et al. [33]. There was 0.01 mL of sample in the assay mixture and 0.12 mL of sodium pyrophosphate buffer (pH 8.3, 0.052 M), 0.01 mL of phenol methosulphate (186 m), and 0.03 mL of NBT (300 m). The NADH was added to start the reaction. After 90 s at 300 °C, the reaction was halted by adding 0.1 mL glacial acetic acid, centrifuged, and separated. The color intensity of the chromogen in the butanol layer was measured at 560 nm against n-butanol. 

#### 2.6.2. Estimation of Glutathione (GSH)

GSH was estimated according to the procedure of Moron et al. [34]. GSH reacts with Ellman’s reagent (5, 5-dithio bis (Nitrobenzoic acid) or DTNB) to produce the chromophore TNB with maximal absorbance at 412 nm as well as oxidized glutathione GSSG. The amount of glutathione measured represents the sum of reduced and oxidized glutathione in the sample. The rate of absorbance change (ΔA 412 nm/min) was made to be linear for the convenience and consistency of measurement, as well as is linearly proportional to the total concentration of GSH. 

#### 2.6.3. Estimation of Catalase (CAT)

Catalase was assayed according to the method of Aebi, [35]. The estimation was done spectrophotometrically following the decrease in absorbance at 230 nm. The blood sample was homogenized in M/150 phosphate buffer (pH 7.0) at 1–4 °C also centrifuged at 5000 rpm. The reaction mixture contained 0.01 M phosphate buffer (pH 7.0) 2 mM H_2_O_2_ and the enzyme extract. The specific activity of Catalase was expressed in terms of units/grams. Absorbance values were compared with a standard curve generated from known catalase. 

#### 2.6.4. Determination of Glutathione Peroxidase (GPx)

GPx was estimated according to the procedure of Moron et al. [34]. The GPx can be measured spectrophotometrically. The 9.02 sodium azide solution, GPx solution and glutathione were added into the beta-NADPH containing vial. We mixed by inversion and pH was adjusted up to 7.0 at 25 °C with 1 M HCl. In the 3.05 mL of reaction mixture, final concentrations were 48 mM sodium phosphate, 0.38 mM ethylenediaminetetraacetic acid, 0.12 mM beta-NADPH, 0.95 mM sodium azide, 3.2 unit of glutathione peroxidase, and 0.075 to 0.15 unit of glutathione, 0.02 mM DL-dithiothreitol, hydrogen peroxide. Absorbance was measured at 340 nm after 5 min.

#### 2.6.5. Determination of Advanced Oxidation Protein Products (AOPPs)

AOPPs were estimated according to the procedure of Kalousová et al. [36]. AOPPs were measured by spectrophotometry on a microplate reader (model MR 5000, Dynatech, Paris, France) and were calibrated with chloramine-T (Sigma, St. Louis, MO, USA) solutions that, in the presence of potassium iodide, absorb at 340 nm. 

#### 2.6.6. Advanced Glycation End-Products (AGEs) Determination

In vitro, AGE-HSA was made by incubating HSA (type V; Sigma; 50 mg/mL) with 500 mM glucose in PBS for 65 days at 37 °C. TCA precipitated plasma proteins or AGE-HSA. It was then dissolved in 250 mL 0.01 M heptafluorobutyric acid (Sigma). Then, 4 mg plasma protein was injected into an HPLC apparatus (Waters Division of Millipore, Marlborough, MA, USA), 30.46 cm C18 Vydac type 218TP (10 mm) (Separations Group, Hesperia, CA, USA). From 0 to 35 min, HPLC was designed with a 10% acetonitrile gradient. Pentosidine was eluted in approximately 30 min using 335 nm excitation and 385 nm emission fluorescence.

#### 2.6.7. Determination of Nitric Oxide (NO)

To make the Griess reagent, combined equal parts N-(1-naphthyl) ethylenediamine and sulfanilic acid. In a spectrophotometer cuvette, mixed 100 mL Griess reagent, 300 mL nitrite-containing sample, and 2.6 mL deionized water. A photometric reference sample was prepared by combining 100 μL Griess reagent with 2.9 mL deionized water (see above) and compared the nitrite-containing sample’s absorbance at 548 nm to the reference sample’s. The absorbance measurements were compared to a known NO standard curve [37].

#### 2.6.8. Estimation of Thiobarbituric Acid Reactive Substances (TBARS)

Lipid peroxidation in blood samples was estimated calorimetrically through measuring thiobarbituric acid reactive substances (TBARS) by the method of Ohkawa et al. [38]. To 0.2 mL of the sample, 0.2 mL of 8.1% sodium dodecyl sulfate (SDS), 1.5 mL of 20% acetic acid, as well as 1.5 mL of 0.8% TBA, were added. After centrifugation at 3000 rpm for 10 min, the upper organic layer was taken and its OD was read at 532 nm against an appropriate blank without the sample. The levels of lipid peroxides were expressed as millimoles of thiobarbituric acid reactive substances (TBARS) per gram of the liver tissue using the standard curve.

Glutamate, homocysteine, acetylcholinesterase (AchE), 4-hydroxynonenal (4-HNE), IsoP-2α, cyclooxygenase-2 (COX-2), tumor growth factor-β (TGF-β), matrix metalloproteinase-6 (MMP-6), interleukins (IL-17, IL-2), CANP, receptor of advanced glycation end-products 9RAGE0, caspase-8, peptidyl arginine deiminase-2 (PAD-2), p38, tumor necrosis factor-alpha (TNF-α) and myelin binding protein (MBP) were analyzed through their corresponding enzyme-linked immune-assay (ELISA) kits using the protocol mentioned in their respective manuals purchased from MyBioSource, San Diego, CA, USA.

### 2.7. Histopathological Examination

The histopathology of the tissue samples (brain, spinal cord, and liver) was performed according to the procedures described by Bancroft [39]. The tissue slides were stained using Hematoxylin and Eosin (H&E) staining procedure. 

### 2.8. Statistical Analysis

The data was interpreted using window-based software SPSS (v.21) and GraphPad Prism (v. 8.0). One-way analysis of variance (ANOVA) followed by Dunnett’s multiple comparison test was performed to determine the statistically significant differences between the means of two or more independent (unrelated) groups with the confidence of 95% (*p* ≤ 0.05). The results were presented as mean ± SD (standard deviation). 

## 3. Results

### 3.1. Body Mass of Animals

**Figure 1:**
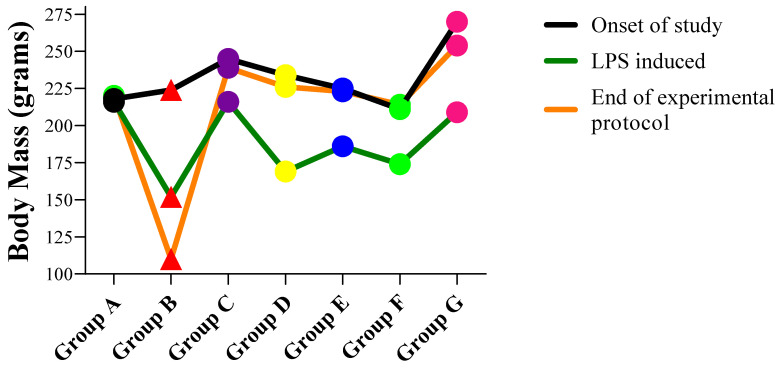
The trend of body mass of Wistar Rats during the experimental protocol.

The body mass of Wistar rats in all groups A, B, C, D, E, F, G before the onset of the study, after the EAE induction, and at the end of the experimental protocol are shown in Figure 1. The body mass decreased notably after the LPS induction which then improved after the treatment was given. 

### 3.2. Inflammatory Markers Profile

**Figure 2:**
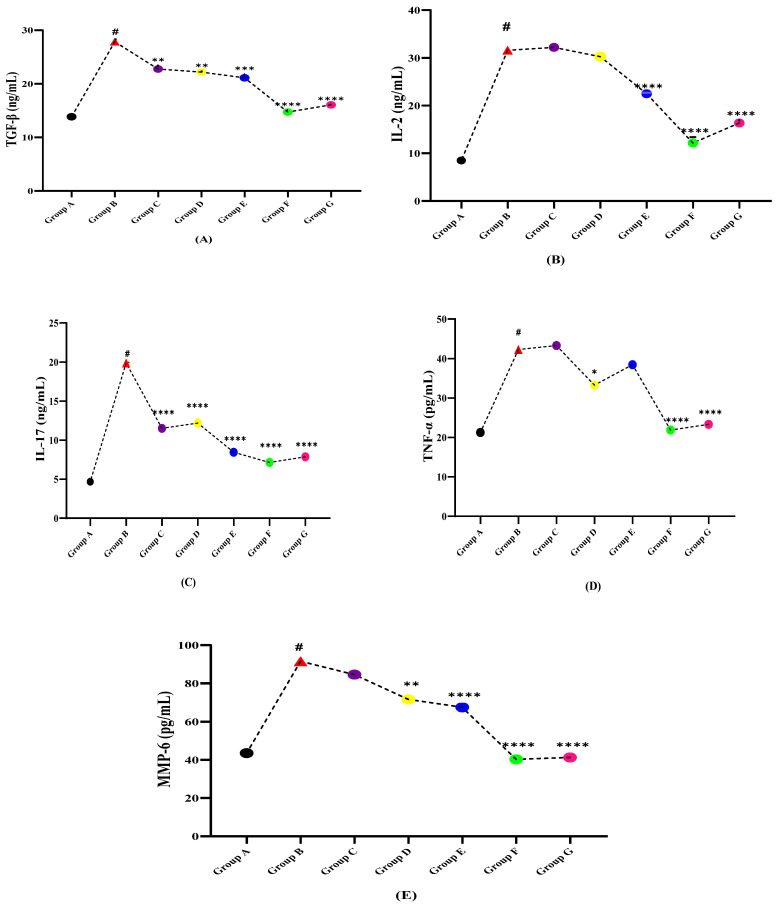
Effect of plant extracts treated groups against LPS induced multiple sclerotic Wistar rats (**A**) TGF-β, (**B**) IL-2, (**C**) IL-17, (**D**) TNF-α, and (**E**) MMP-6. (**#** indicate reference group EAE model while Asterisks are showing the significance in the improvement of results as compared to reference group B). (*) slight significance, (**) moderate significance, (***) high significance, (****) very highly/effective significance.

The levels of cytokines TGF-β, IL-2, IL-17, TNF-α, and MMP-6 in normal, diseased, and treatment groups are shown in Table 2. The serum levels of all the aforesaid variables were found to remarkably increase on disease induction via LPS as compared to the normal control group A due to triggered inflammatory response. Whereas, the treatment groups (C, D, and E) given independent extracts of *N. hindustana*, *V. negundo*, and *A. albiflora* and their cocktail (Group F) dose significantly improved the levels (*p* > 0.0001) showing the notable effect of extracts on inflammatory cytokines. Figure 2A–E shows the effectiveness of plant extracts treated groups in restoring the variables as compared to LPS induced MS Wistar rat group B and displays that although all treatments showed varying significance, the communal dose of all three selected plants (Group F) has proven significant effect (*p* < 0.0001) in restoring the disease as compared to the dose of plant extract separately and is comparable with the standard drug Nimodipine given (Group G). 

### 3.3. Circulatory Stress Markers Profile

**Figure 3:**
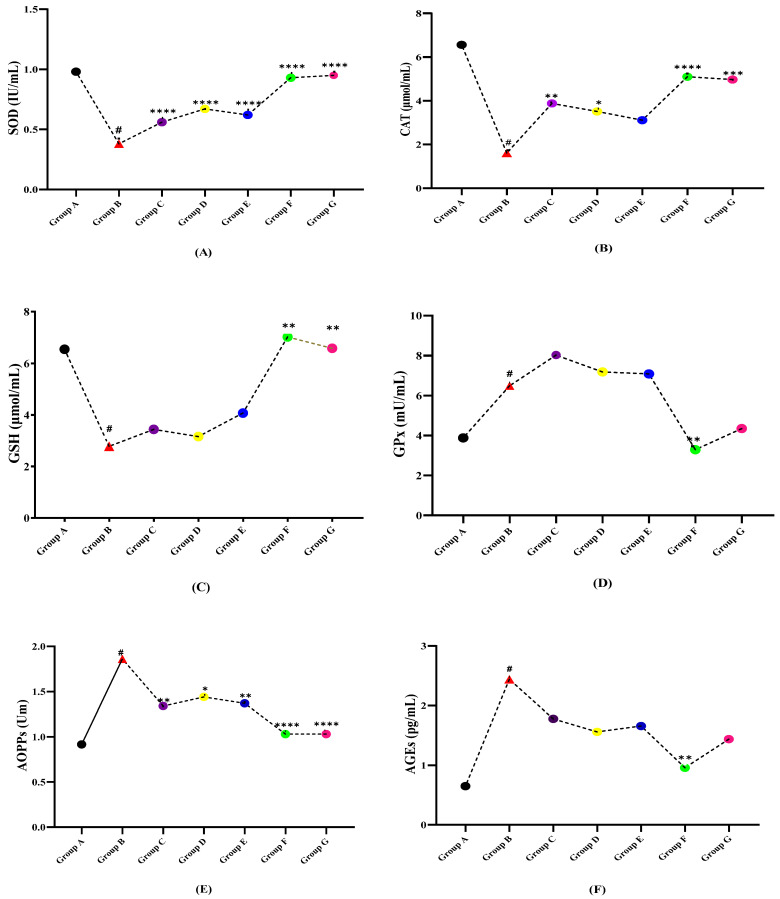

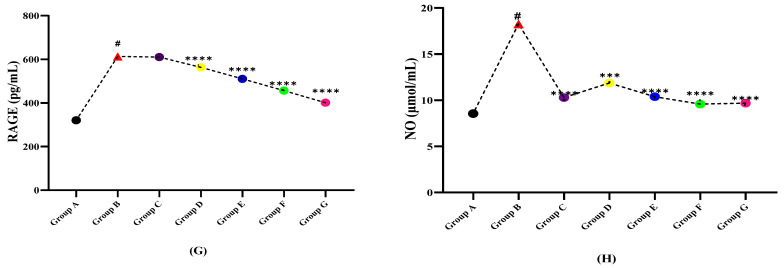
Comparison of plant extracts treated groups independently against LPS induced multiple sclerotic Wistar rats (**A**) SOD, (**B**) CAT, (**C**) GSH, (**D**) GPx, (**E**) AOPPs, (**F**) AGEs (**G**) RAGE, and (**H**) NO. (# indicate reference group EAE model while Asterisks are showing the significance in the improvement of results as compared to reference group B). (*) slight significance, (**) moderate significance, (***) high significance, (****) very highly/effective significance.

By the end of the experimental protocol, interesting and significant trends among levels of anti-oxidant markers (SOD, CAT, GSH, GPx) activities in multiple sclerosis and the treatment groups were observed (Table 3). The levels of SOD, CAT, and GSH were found to be significantly higher in the control healthy group A. After LPS induction, these seemed to be compromised due to disease induction and an excess of free radicals’ production while the GPx levels (6.49 ± 2.88 mU/mL) showed an inverse action. In treatment groups (C, D, E, F, G), upgradation in the level of all antioxidants (SOD, CAT, GSH) was observed. While compared with diseased group B as pictured in Figure 3A–D, among all of the treatment groups, the combined doses of all three plant extracts showed significant recovery of antioxidant levels as compared to the dose given with a single plant extract which can be considered as an alternate for the given standard drug. The results of oxidative stress variables including advanced oxidation protein products (AOPPs), and advanced glycation end-products (AGEs) along with RAGE, and nitric oxide (NO) were influenced by the disease. The levels of AOPPs, AGEs, RAGE, and NO in LPS induced group B were observed to be high which may be due to excessive formation of oxidative free radicals that in turn, cause the oxidation of macromolecules. While in normal group A and all treatment groups (C, D, E, F, G), the levels of AOPPs, AGEs, RAGE, and NO were shown to be significantly (*p* < 0.0001) lower. As shown in Figure 3, the AOPPs, RAGE, and NO reported high significance as compared to CAT and GPx in all treated groups. Furthermore, the cocktail extract dose due to synergistic effects showed great effectiveness in restoring antioxidant levels and decline OS markers in comparison with the diseased group B.

### 3.4. DNA Damage Profile

**Figure 4:**
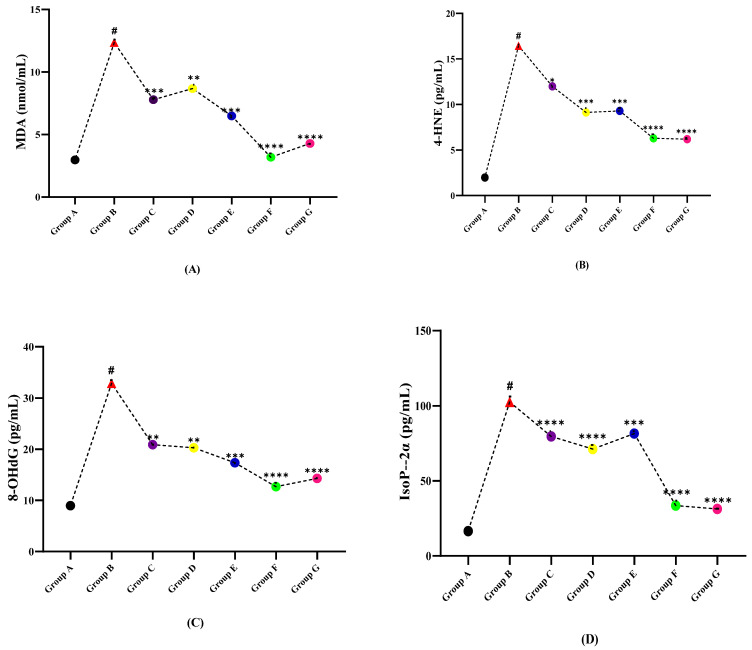
Comparison of plant extracts treated groups independently against LPS induced multiple sclerotic Wistar rats (**A**) MDA, (**B**) 4-HNE, (**C**) 8-OHdG, and (**D**) Iso5P-2α. (# indicate reference group EAE model while Asterisks are showing the significance in the improvement of results as compared to reference group B). (*) slight significance, (**) moderate significance, (***) high significance, (****) very highly/effective significance.

The data assembled in Table 4 illustrate that the level of MDA in healthy group A is the lowest while the LPS induced group B has the highest value. However, after treating with different doses of plant extracts, significant declining effects on the level of MDA were observed. The DNA damage markers 4HNE, IsoP-2α, and 8-OHdG levels were found low in the control group, which after LPS induced EAE (group B), were raised. While a significant reduction in the levels of these markers was observed when treated with plant extracts. The results of DNA damage parameters have depicted (Figure 4) that, the damage induced by LPS is significantly reduced by the different extracts of plants. However, the combined extract dose (Group F) seemed to work best in restoring the DNA damage and is comparable with the standard drug nimodipine (Group G).

### 3.5. Receptor Signaling Profile

**Figure 5:**
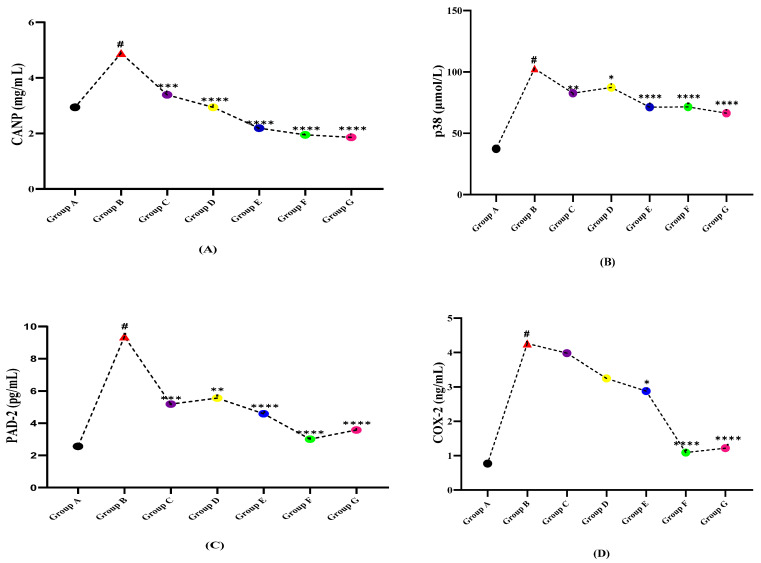

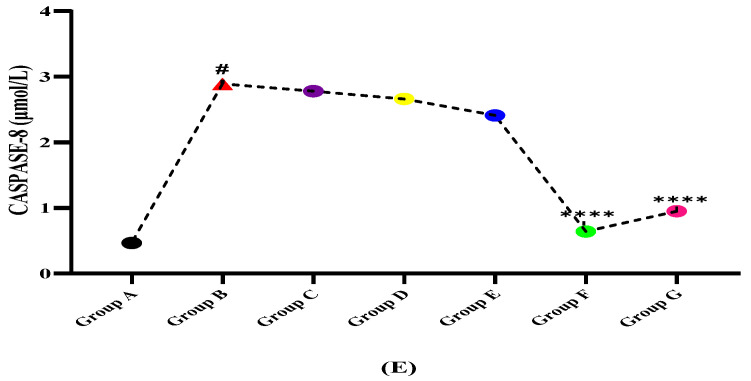
Comparison of plant extracts treated groups independently against LPS induced multiple sclerotic Wistar rats (**A**) CANP, (**B**) p38, (**C**) PAD-2, (**D**) COX-2, and (**E**) caspase-8. (# indicate reference group EAE model while Asterisks are showing the significance in the improvement of results as compared to reference group B). (*) slight significance, (**) moderate significance, (***) high significance, (****) very highly/effective significance.

The fluctuation of molecular markers including CANP, PAD-2, p38, caspase-8, and COX-2 was analyzed in the serum of both normal and LPS induced rats (Table 5, Figure 5). The serum levels of CANP and caspase-8 in healthy rat group A were found to be 2.94 ± 0.556 mg/mL and 0.465 ± 0.02 µmol/L. In LPS induced group B, it was found to be 4.89 ± 0.956 mg/mL and 2.896 ± 0.99 µmol/L, respectively, while, in treatment given groups C, D, E, F, G their levels seemed to have a declining trend. The PAD-2 serum levels in all of the seven groups (A, B, C, D, E, F, G) were found to be 2.56 ± 0.19 pg/mL, 9.37 ± 2.35 pg/mL, 5.19 ± 1.04 pg/mL, 5.56 ± 2.19 pg/mL, 4.59 ± 0.94 pg/mL, 3.01 ± 0.98 pg/mL, and 3.58 ± 1.19 pg/mL, respectively (Table 4). In isolated treatment groups, *A. albiflora* ethanolic extract-treated group E has shown more influential effects as compared to *N. hindustana* treated group C and *V.negundo* treated group D while the combination extracts group (group F) seemed to be lower as compared to standard drug group G. The levels of p38 and COX-2 were observed to bee minimal in normal healthy control group A and in the diseased group B, their levels were found aggravated while, after treatments with selected plant extracts and standard drugs, their levels reduced in groups C, D, E, F, G. Among the observed diminishing levels of CANP, caspase-8, PAD-2, p38, and COX-2 with the plant extract treated groups, the combination extracts dose (Group F) shows more significant results as compared to the single extract treatment groups (Group C, D, E) except p38 in which *A.albiflora* extract dose (group E) shows more vibrant results as compared to combined extract dose (Group F). In comparing the LPS induced MS group vs. plant extract treatment groups, the synergistic dose showed more effectiveness in all variables except COX-2 (Figure 5).

### 3.6. Protein and Amino Acid Profile

**Figure 6:**
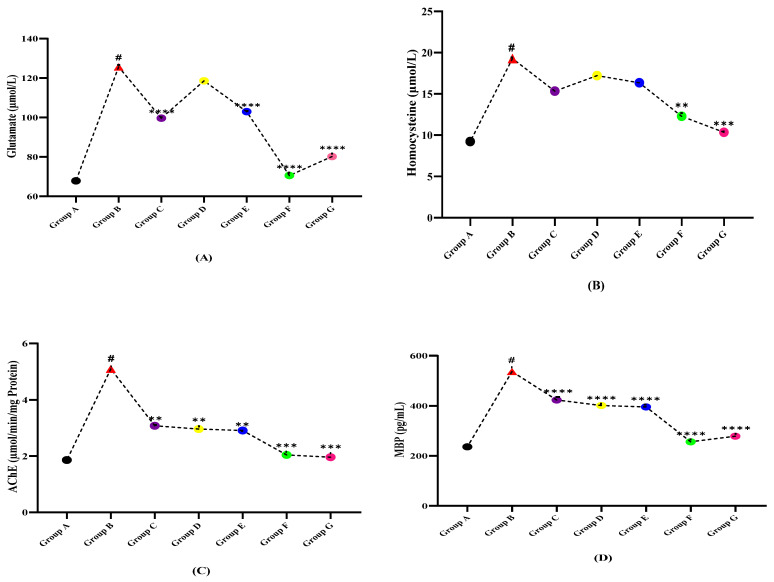
Comparison of plant extracts treated groups independently against LPS induced multiple sclerotic Wistar rats (**A**) glutamate, (**B**) homocysteine, (**C**) acetylcholinesterase (AChE), and (**D**) myelin binding protein (MBP). (# indicate reference group EAE model while Asterisks are showing the significance in the improvement of results as compared to reference group B). (*) slight significance, (**) moderate significance, (***) high significance, (****) very highly/effective significance.

The results of protein and amino acid markers including homocysteine, glutamate, homocysteine, acetylcholinesterase (AChE), and MBP (myelin binding protein) illustrated in Table 6 and Figure 6 show that the fluctuated levels of amino acids and proteins might play an important role in the development of multiple sclerosis and the selected plant treatment displayed a diverse role as therapeutic agents in multiple sclerosis. As shown in Table 6, the level of amino acids (glutamate and homocysteine) were raised on the LPS dose given, while when treated with plant extract dose, it normalized their aggravated levels. Myelin binding protein (MBP) is increased in EAE due to demyelination of neurons while the given plant extracts have reduced showing their role in remyelination.

### 3.7. Histopathological Evaluation

#### 3.7.1. Brain

**Figure 7:**
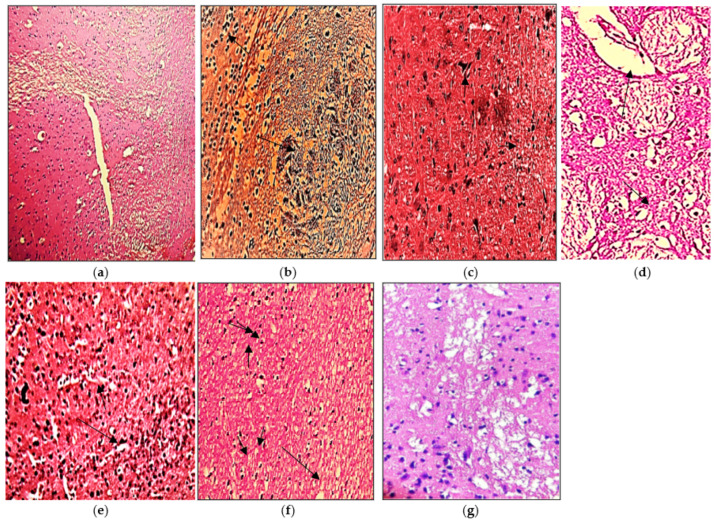
Pictorial presentation of brain H&E stained sections: (**a**) normal control group rat, (**b**) LPS intoxicated rat brain, (**c**) *N. hindustana* extract-treated group, (**d**) *V. negundo* treated group, (**e**) *A. albiflora* ethanolic extract-treated group, (**f**) conjugation of *N. hindustana*, *V. negundo*, and *A. albiflora* treated group, (**g**) brain section of the standard drug group. Objective size is 40×. Arrows represent edematous plaques, sparse lobular lymphoplasmacytic infiltration, and marked demyelination.

The histopathological presentation of the brain also supported the protective effect of the extracts compared to the LPS induced disease groups as shown in Figure 7. Microscopic analysis of normal oligodendrocytes shows the absence of microscopic lesions and microglial inactivation (Figure 7a). Moreover, cerebral folia are observed to be intact with no excessive infiltration. However, inflamed brain cells by lipopolysaccharides illustrate the presence of multiple focal areas of myelin loss with localized multinucleated Creuzfeldt cells (Arrow) (Figure 7b). Edematous plaques, sparse lobular lymphoplasmacytic infiltration, and marked demyelination are the hallmark of multiple sclerosis. The treated cells with ethanolic extract of *N. hindustana* expressed 65% recovery with moderate necrosis of the white matter having demyelinated oligodendrocytes which were seen to be more frequent in areas with macrophages than the areas lacking macrophages. Gliotic scar in inactive lesions is due to the presence of astrocytes but malignant changes are not seen as presented in Figure 7c. The cells treated with ethanolic extract of *V. negundo* demonstrate several folia in the cerebellum as shown in normal cells (Figure 7d). The characteristic features of cerebellar folium with external germinal layer, molecular layer, Purkinje cell layer, and granular cell layer were seen which depicts 45% recovery. Scattered dark stained Purkinje cells were more pronounced in neurons, molecular and granular layers. Microscopic lesions of dendritic cells treated with *A. albiflora* extract showed fused stellate cells with an area of little demyelination consisting of necrotic fibers with 60% myelination in axons (Figure 7e). The experiment depicted optimal myelination of dendritic neurons treated with the conjugated extract of *N. hindustana*, *V. negundo*, and *A. albiflora* with 86.7% reduced degeneration of neuronal sheath along with the atrophied area of neuroglial cells (Figure 7f). The standard Nimodipine treated cells in Figure 7g show remyelination of axonal terminals with no degeneration of oligodendrocytes.

#### 3.7.2. Liver

**Figure 8:**
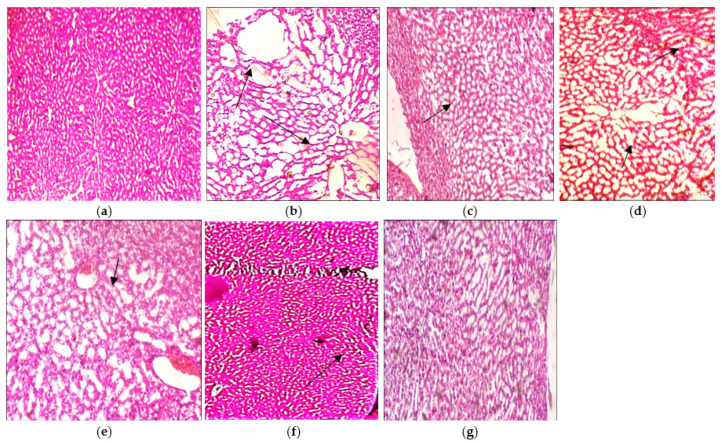
Histopathological results of liver tissues: (**a**) normal control group rat, (**b**) LPS intoxicated rat liver, (**c**) *N. hindustana* extract-treated group, (**d**) *V. negundo* treated group, (**e**) *A. albiflora* ethanolic extract-treated group, (**f**) conjugation of *N. hindustana*, *V. negundo*, and *A. albiflora* treated group, (**g**) brain section of the standard drug group. Objective size is 40×. Arrows represent mononuclear cell infiltration with congested sinusoids due to the enlargement of hepatic capillaries.

The histopathological interpretation of the liver also supported the protective effect of the plants’ extracts compared to the LPS induced disease groups (Figure 8). Microscopic analysis of normal hepatocytes reveals the normal architecture of hepatocellular fatty vacuoles with sinusoids (Figure 8a). Rats receiving an injection of LPS disclose pronounced mononuclear cell infiltration with congested sinusoids which is due to the enlargement of hepatic capillaries (Figure 8b). Rats receiving *N. hindustana* ethanolic extract expressed 60% recovery and the hepatic hemorrhages along with binucleated hepatocytes were visible in some areas. Mild degenerative changes in hepatocytes with abnormal hepatic cords were evident in hepatic parenchyma as presented in Figure 8c. Rats receiving *V. negundo* ethanol extract have shown the development of vacuolation in hepatocytes away from the central vein (Figure 8d). Disarray in lobular hepatic structure is due to inflammation and injury which reveals 40% recovery. A 50% recovery was shown with mild hemorrhages in the focal areas with Karyolysis detected in some hepatocytes. There was mild hepatic congestion with disrupted endothelial lining of the central vein in rats receiving *A. albiflora* extract treatment as presented in Figure 8e. When the rats were injected with the cocktail of three plant extracts, 70% of recovery was observed (Figure 8f), which indicates disruption of the central vein with leakage of blood sinusoids and the influx of inflammatory cells. Fatty changes in the hepatocytes were pronounced with visible Pyknotic nuclei. The standard Nimodipine treated the cells in Figure 8g reveals regenerated in necrotic hepatocytes with pyknotic nuclei seen.

#### 3.7.3. Spinal Cord

**Figure 9:**
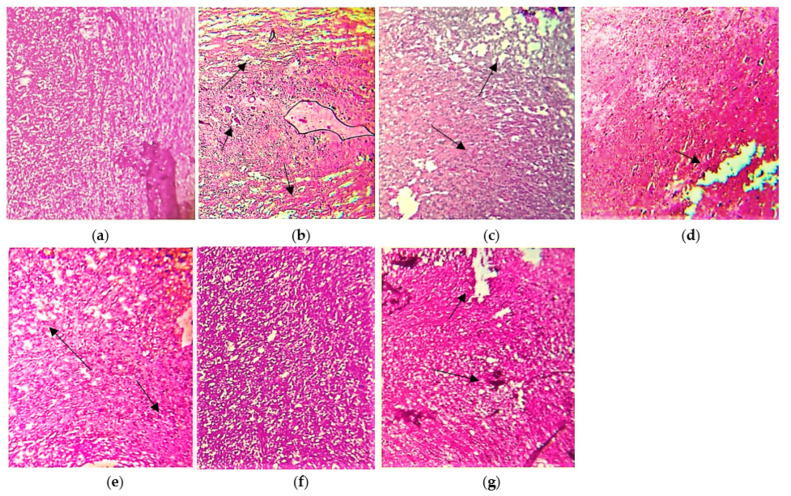
Histopathological outcomes of spinal cord tissues: (**a**) normal control group rat, (**b**) LPS intoxicated rat spinal cord tissues, (**c**) *N. hindustana* extract-treated group, (**d**) *V. negundo* treated group, (**e**) *A. albiflora* ethanolic extract-treated group, (**f**) conjugation of *N. hindustana*, *V. negundo*, and *A. albiflora* treated group, (**g**) brain section of the standard drug group. Objective size is 40×. Arrows represent pronounced hypercellular lesions due to inflammatory cells infiltration and demyelination.

The histopathological evaluation of spinal cord tissues also supported the protective effect of these extracts when compared to the LPS induced disease groups as shown in Figure 9. Microscopic analysis of the normal white matter of the spinal cord reveals normal architecture (Figure 9a)**.** Rats receiving an injection of LPS disclose pronounced hypercellular lesions due to inflammatory cells infiltration and demyelination as shown in Figure 9b. Rats receiving ethanolic extracts *N. hindustana*, *V. negundo*, and *A. albiflora* expressed a nearly similar percentage of recovery with mild degeneration and asymmetry in structure due to inflammation and injury as portrayed in Figure 9c–e. The histopathological interpretation of the cocktail of three plant extracts treated group F depicts an average recovery of 75% indicating the reversal of dysmyelinated areas along with some structural symmetry (Figure 9f). The standard Nimodipine treated cells reveal slight regeneration in necrotic areas as shown in Figure 9g.

## 4. Discussion

The present study investigates the effectivity as well as anti-inflammatory, anti-oxidative, and neuro-pharmacological properties of various extracts of *N. hindustana*, *V. negundo*, and *A. albiflora* plants in LPS induced rat model. LPS induces inflammation in the brain, accompanied by neuronal loss and activation of microglia, that induced the release of various inflammatory cytokines through NF-kB signaling pathway activation [40]. This supports the finding of the current study representing the raised inflammatory markers after giving the injection of LPS to Wistar rats. MS is primarily instigated due to immune-mediated inflammation along with neurodegenerative progression [41]. The p38 MAPK pathway has a tremendous role in the regulation of inflammatory responses as it promotes the production of many T-cell polarizing and pro-inflammatory cytokines including IL-17, TNF-α. Inflammation might raise the ROS levels that could lead to oxidative stress. Oxidative stress, energy failure, and mitochondrial injury can be linked with neurodegeneration as well as plaque formation in lesions of gray and white matter [42,43]. Pro-inflammatory cells, primarily activated macrophages, are key organizers of systemic inflammatory response syndrome and are responsible for the majority of cellular and molecular pathophysiology through cytokines such as TNF-α, IL- (2,6,8,12,17) as well as free radicals [44,45]. The current study, investigates the influence of selected plant extracts independently as well as their communal dose which shows the reduction in abrupt and excessive inflammatory markers response including TNF-α, IL-2, and TGF-β, significantly. This reduction in inflammatory markers might be due to the presence of active phytoconstituents having anti-inflammatory properties.

Inflammation-induced NO overproduction was primarily mediated by inducible nitric oxide synthase (iNOS), which was up-regulated in macrophages following LPS stimulation [46]. Inflammation similarly caused increased COX-2 expression when iNOS expression was raised in inflammatory cells [47]. COX-2 expression is reported to be dependent on NO levels, and iNOS binds to, s-nitrosylates and activates COX-2 [48]. Mitogen-activated protein kinases (MAPKs), such as p38, can mediate LPS-induced iNOS and COX-2 gene expression and synthesis [49,50]. The presence of free radicals in nervous tissues might be toxic like peroxy-nitrite that can enhance inflammatory response hence supporting the results of the present study. LPS also increases the levels of oxidative stress markers including NO [51]. NO participates in the development of MS through the breakdown of BBB also through direct damage of tissues, plaque formation, blockage of axonal conduction, and axonal degeneration [52]. We have also observed excessive NO and COX-2 production on LPS induction that was observed reduced after selected plant treatment. Lipid peroxidation may activate the apoptosis process by stimulating intrinsic pathways that exist in all cells [53]. Redox reaction enhances the activity of matrix metalloproteinases (MMPs) that play an important part in the trafficking of T-cells into the central nervous system [54,55]. 4-hydroxyalkenals and MDA are copious diffusible products considered to take part in harmful effects of the lipid peroxidation significantly in the tissues as well as in serum. The reactive aldehydes of lipid peroxidation repel some cellular nucleophiles involving lipids, proteins, and nucleic acids [56]. MDA, NO, MMP-6, neutrophil collagenase, and 8-OHdG are important biomarkers for oxidative stress and DNA damage. MDA, NO, MMP-6, and 8-OHdG concentrations increased in serum of diseased rats (compared with healthy controls) treated with LPS endotoxin inducing superoxide’s oxidative stress in the neuronal cells in the brain and spinal cord. The extracts cocktail of all three plants significantly reduces the levels of MDA, NO, MMP-6, and 8-OHdG showing the reduction in oxidative stress and highlighting its neuroprotective role. The current research has depicted that AOPPs and AGEs were produced during the stressed conditions and accountable to prompt the inflammatory process have shown aggravated levels in the diseased induced group that could be linked to disease progression while, treating these induced groups with different extracts, we found these oxidative macromolecules end products in lower concentration hence, reflecting with the finding of Oliveras-Lopez et al., [57]; Gonzalo-Goernado et al. [58].

The oxidative stress and loss of antioxidant balance lead to consequences of the disease. SOD, CAT, and glutathione peroxidase are primary antioxidants involved in the direct ROS mechanism while glutathione is considered as a secondary antioxidant that cooperates in the ROS detoxification via reducing peroxide levels [59,60]. The current study has reported that the levels of various antioxidants including SOD, CAT, GSH have been drastically decreased at disease induction in all groups as evident from previous studies [61,62] and their levels were significantly restored after treatment with the selected plant extracts which is concurrent with the study of Kumar et al. [63]. GPx in the MS individual’s erythrocytes continues undistinguishable meanwhile contradictory data about its activity [64]. Glutathione-peroxidase is known as a free radical tracker enzyme and plays a vital role in the defense system of antioxidants. Increased activity of glutathione-peroxidase present in the MS patient’s serum might be responsible for minimizing the cell injury due to oxidative stress. When LPS induced diseased rats were treated with Nimodipine, and various extracts of *N. hindustana*, *V. negundo*, and *A. albiflora* and their cocktail, the content of GSH, CAT, and SOD were significantly improved, thus reducing the oxidative load of free radicals. These extracts showed more effective results than the conventional drug, while their cocktail showed a synergic effect.

The fragments of central inflammation are accountable for the irregular relapsing-remitting stage of MS, while axonal loss and neurodegeneration might be responsible for the progressive symptoms that may cause disability [65]. The EAE rats presented the clinical signs and symptoms including weight loss, limb paralysis, and fur tarnishing as well as diminished neuronal density that may cause neuronal death via necrosis and apoptosis. These changes were corroborated by high caspases and LDH levels in the blood and brain [66]. The current study reported the increased levels of caspases-8 in LPS induced MS rats which were observed reduced after treatment with selected plant extracts. This might be due to the reason that caspase-8 is predominantly expressed by microglia, and regulates the central nervous system (CNS) immunity. Caspases-8, in particular, is an extrinsic apoptotic pathway activated via various TNF-family receptors at the plasma membrane causing receptor clustering which in turn, involve in the recruitment of Fas-associated death protein (FADD) that binds death effector domains (DEDs) in the caspase-8 leading cleavage and initiates apoptosis [65]. Caspase-8 is a link between various death mechanisms including ER stress, autophagic pathways as well as many intrinsic and extrinsic pathways [67]. Pathomechanisms of excite-toxicity are linked with calcium overload, dysfunction of ion channels, glutamate overload [68], apoptotic pathway activation, mitochondriopathies, and production of proteolytic enzymes. Neurotransmitter glutamate showed increased levels in CSF, CNS, and peripheral blood as well, in acute lesions of the MS as well as normal-appearing white matter (NAWM) in individuals [69]. The study Poddighe et al. [70] reported that MS suffering people had increased plasma L-glutamate levels which support the finding of the current study.

PAD-2 was present in high quantity in MS patients as compared to other neuropathologies [71,72,73]. MBP is considered the best PAD-2 substrate and the decreased positive charge on MBP leads to citrullination reaction, destabilizing the lipid bilayer and myelin interaction, which affects myelin structure and makes it vulnerable to various proteases [74]. Citrullinated MBP can induce and sustain inflammation in EAE [75]. The expression of MBP is increased in EAE [76]. In the current study, the levels of PAD-2 and MBP are significantly observed higher on LPS induced EAE, which were reduced after being given selected plant extract treatments showing their involvement in the remyelination process.

Histopathological analysis showed supportive results for the biochemical markers. The brain, spinal cord, and liver of LPS induced Wistar rats showed necrosis, inflammation, and loss of symmetry in respective organ cells. While the plant extract treatment group showed noteworthy repair in architecture as well as reverse oxidative stress in the brain and spinal cord relative to the normal healthy groups. The combined plant extract dose of all selected plants exhibits the most significant findings.

## 5. Conclusions

MS as being a multifactorial autoimmune disease including the deterioration of neurological function that involves oxidative stress, inflammatory cytokines, and antioxidant downfall which in turn, may cause molecular and DNA damage leading to immune regulatory defects such as paraparesis. The present study for the first time found clinical evidence of *Nepeta hindustana*, *Vitex negundo*, and *Argemone albiflora* against multiple sclerosis. The results of the current study show that these plants have neuroprotective, antioxidant, and anti-inflammatory phytochemicals and secondary metabolites, which exhibit substantial effects on remyelination and neuroprotection through the suppression of stress markers, which repair the molecular as well as DNA damage. Our results also conclude that in the future, these plants may be used as potential natural neuro-therapeutic candidates against multiple sclerosis. We further recommend a comprehensive study in this area to isolate the exact active compounds present in these extracts for optimal composition responsible for the therapeutic purpose.

## Figures and Tables

**Table 1:** Experimental design for disease induction and treatment protocol.

Groups	Treatments
A	CONTROL
B	LPS
C	LPS + *Nepeta hindustana*
D	LPS + *Argemone albiflora*
E	LPS + *Vitex negundo*
F	LPS + *Nepeta hindustana+ Argemone albiflora+ Vitex negundo*
G	LPS + Nimodipine^®^

Nepeta Hindustana (NH), Vitex Negundo (VN), Argemone Albeflora (AA) for 28 days (400 mg/Kg B.Wt/day). NIMODIPINE^®^ @ (30 mg/Kg B.Wt/day) for 28 days.

**Table 2:** Inflammatory Markers Profile Of LPS Induced Multiple Sclerotic Wistar Rats.

Groups	Mean ± SD (n = 5)				
	TGF-β (ng/mL)	IL-2 (ng/mL)	IL-17 (ng/mL)	TNF-α (pg/mL)	MMP-6 (pg/mL)
A	13.86 ± 2.80	8.49 ± 1.09	4.68 ± 1.39	21.25 ± 4.19	43.56 ± 5.19
B	27.84 ± 1.23	31.59 ± 2.19	19.89 ± 3.08	42.26 ± 7.45	91.58 ± 6.23
C	22.77 ± 2.61	32.19 ± 1.88	11.51 ± 1.36	43.29 ± 2.99	84.59 ± 4.77
D	22.19 ± 2.67	30.25 ± 3.19	12.19 ± 2.16	33.29 ± 4.47	71.59 ± 4.19
E	21.12 ± 2.06	22.49 ± 3.55	8.45 ± 0.16	38.47 ± 2.99	67.49 ± 7.77
F	14.73 ± 1.58	12.16 ± 4.29	7.15 ± 1.76	21.88 ± 4.17	40.29 ± 5.66
G	16.08 ± 1.05	16.35 ± 1.18	7.87 ± 1.14	23.33 ± 3.77	41.29 ± 8.47
*p*-Value	<0.0001	<0.0001	<0.0001	<0.0001	<0.0001

**Table 3:** Circulatory Stress Markers Profile Of LPS Induced Multiple Sclerotic Wistar Rats.

Groups	Mean ± SD (n = 5)							
	SOD IU/mL	CAT µmol/mL of Prot.	GSH µmol/mL	GPX mU/mL	AOPPs (Um)	AGEs pg/mL	RAGE pg/mL	NO (µmol/L)
A	0.98 ± 0.01	6.56 ± 1.69	6.54 ± 1.89	3.88 ± 0.87	0.916 ± 0.052	0.65 ± 0.04	320.56 ± 12.09	8.55 ± 2.55
B	0.38 ± 0.047	1.61 ± 0.78	2.78 ± 0.99	6.49 ± 2.88	1.86 ± 0.12	2.44 ± 0.69	613.08 ± 18.33	18.26 ± 2.29
C	0.56 ± 0.038	3.88 ± 0.89	3.44 ± 0.99	8.03 ± 0.17	1.34 ± 0.23	1.78 ± 0.62	610.32 ± 17.59	10.29 ± 1.26
D	0.67 ± 0.008	3.51 ± 0.65	3.16 ± 1.22	7.19 ± 0.23	1.44 ± 0.08	1.56 ± 0.43	563.26 ± 10.26	11.86 ± 2.19
E	0.62 ± 0.045	3.11 ± 0.56	4.07 ± 2.00	7.09 ± 1.14	1.37 ± 0.21	1.66 ± 0.95	510.23 ± 11.17	10.39 ± 2.56
F	0.93 ± 0.49	5.10 ± 1.08	7.02 ± 1.88	3.29 ± 0.56	1.03 ± 0.24	0.96 ± 0.66	456.35 ± 14.26	9.59 ± 2.87
G	0.95 ± 0.07	4.97 ± 0.56	6.58 ± 2.19	4.35 ± 1.55	1.03 ± 0.34	1.44 ± 0.78	401.26 ± 17.26	9.68 ± 2.19
*p*-Value	<0.0001	<0.0001	<0.0003	<0.0001	<0.0001	0.0058	<0.0001	<0.0001

**Table 4:** DNA Damage Profile Of LPS Induced Multiple Sclerotic Wistar Rats.

Groups	Mean ± SD (n = 5)			
	MDA (nmol/mL)	4-HNE (ng/L)	8-OHdG (pg/mL)	IsoP-2α (pg/mL)
A	2.97 ± 0.63	1.98 ± 0.28	8.96 ± 1.98	16.58 ± 4.17
B	12.35 ± 1.99	16.44 ± 3.66	32.88 ± 6.65	102.58 ± 5.65
C	7.78 ± 2.78	15.99 ± 1.59	30.89 ± 4.77	99.65 ± 4.33
D	8.67 ± 1.97	15.10 ± 2.88	30.29 ± 3.19	71.29 ± 5.47
E	6.48 ± 1.25	14.29 ± 3.19	27.35 ± 8.49	81.65 ± 12.48
F	3.18 ± 0.65	6.29 ± 2.19	12.68 ± 4.07	22.59 ± 7.08
G	4.28 ± 0.71	7.19 ± 1.77	14.29 ± 3.77	31.29 ± 4.77
*p*-Value	<0.0001	<0.0001	<0.0001	<0.0001

**Table 5:** Signaling Profile Of LPS Induced Multiple Sclerotic Wistar Rats.

Groups	Mean ± SD (n = 5)				
	CANP (mg/mL)	p38 (µmol/L)	PAD2 (ng/mL)	Caspase-8 (µmol/L)	COX-2 (ng/mL)
A	2.94 ± 0.556	37.29 ± 3.22	2.56 ± 0.19	0.465 ± 0.02	0.77 ± 0.06
B	4.89 ± 0.956	102.65 ± 10.25	9.37 ± 2.35	2.896 ± 0.99	4.26 ± 1.08
C	3.39 ± 0.24	82.65 ± 12.25	5.19 ± 1.04	2.78 ± 0.64	3.98 ± 1.22
D	2.94 ± 0.18	87.29 ± 4.56	5.56 ± 2.19	2.66 ± 0.54	3.25 ± 0.56
E	2.19 ± 0.42	71.23 ± 8.49	4.59 ± 0.94	2.41 ± 0.27	2.88 ± 0.66
F	1.95 ± 0.14	71.49 ± 8.49	3.01 ± 0.98	0.641 ± 0.095	1.09 ± 0.03
G	1.86 ± 0.23	66.35 ± 9.78	3.58 ± 1.19	0.95 ± 0.09	1.22 ± 0.07
*p*-value	<0.0001	<0.0001	<0.0001	<0.0001	<0.0001

**Table 6:** Protein Profile Of LPS Induced Multiple Sclerotic Wistar Rats.

Groups	Mean ± SD (n = 5)			
	Glutamate (µmol/L)	Homocysteine (µmol/L)	AChE (µmol/min/mg Protein)	MBP (pg/mL)
A	67.88 ± 4.56	9.23 ± 2.11	1.86 ± 0.95	236.26 ± 5.26
B	125.66 ± 7.88	17.28 ± 3.16	3.10 ± 0.68	537.23 ± 16.35
C	118.59 ± 9.18	17.22 ± 3.19	3.08 ± 0.81	423.25 ± 12.26
D	99.65 ± 5.19	16.35 ± 4.26	2.96 ± 0.55	401.25 ± 7.16
E	102.99 ± 4.56	15.34 ± 3.13	2.61 ± 0.88	395.65 ± 11.05
F	70.59 ± 7.19	10.29 ± 2.11	2.04 ± 0.21	256.34 ± 8.19
G	80.19 ± 6.47	16.35 ± 3.19	1.96 ± 0.09	278.65 ± 7.18
*p*-value	<0.0001	<0.0001	<0.0001	<0.0001

## Data Availability

The data presented in this study are available on request from the corresponding author.

## Sample Availability

Samples of the compounds are not available from the first author.
